# Supplementary material for: Non-Invasive vs. Invasive Markers in Ulcerative Colitis: A Systematic Review of Intestinal Ultrasound, Biopsy, and Faecal Calprotectin
Source: Int J Mol Sci. 2025 Aug 22;26(17):8129. doi: 10.3390/ijms26178129 (PMC12428742; doi:10.3390/ijms26178129)
Supplement: Supplementary file 1 [file ijms-26-08129-s001.zip › Supplementary table S1.pdf]

Supplementary table S1. Description of excluded records

Year	Exclusion reason	Authors	Title
**2024**	**Poster presentation**	**Thornton S et al**	1 year follow-up of pediatric ulcerative colitis using intestinal ultrasound
**2024**	**Poster presentation**	**Dolinger MT et al**	30 EARLY INTESTINAL ULTRASOUND RESPONSE TO BIOLOGIC AND SMALL MOLECULE THERAPY PREDICTS ENDOSCOPIC OUTCOMES IN CHILDREN WITH ULCERATIVE COLITIS
**2016**	**Poster presentation**	**Oliva S et al**	A noninvasive follow-up of pediatric ulcerative colitis by using colon capsule endoscopy and ultrasonography
**2015**	**Poster presentation**	**Dell'Era A et al**	Accuracy of fecal calprotectin, bowel ultrasonography and inflammatory indexes in the diagnosis of paediatric inflammatory bowel disease
**2023**	**Wrong outcome**	**Jimbo K et al**	Accuracy of Transperineal Ultrasonography for Assessing Rectal Lesions in Paediatric Ulcerative Colitis: A Prospective Study.
**2025**	**Wrong outcome**	**Innocenti T et al**	Applicability of the International Bowel Ultrasound Segmental Activity Score (IBUS-SAS) to Ulcerative Colitis: A Preliminary Study
**2022**	**Poster presentation**	**Shibuya N et al**	ASSESSMENT OF COLON WALL THICKENING BY INTESTINAL ULTRASOUND IS USEFUL TO DETECT ENDOSCOPIC DISEASE ACTIVITY IN PATIENTS WITH ULCERATIVE COLITIS

**2021**	**Poster presentation**	**De Voogd F et al**	Baseline hypertrophy of the submucosa at intestinal ultrasound predicts failure of treatment in patients with ulcerative colitis
**2023**	**Wrong outcome**	**St-Pierre J et al**	Bedside Intestinal Ultrasound Performed in an Inflammatory Bowel Disease Urgent Assessment Clinic Improves Clinical Decision-Making and Resource Utilization.
**2024**	**Wrong outcome**	**Chavannes M**	Bedside Intestinal Ultrasound Predicts Disease Severity and the Disease Distribution of Pediatric Patients With Inflammatory Bowel Disease: A Pilot Cross-sectional Study
**2023**	**Poster presentation**	**Les A et al.**	Bowel Doppler Signal, the most important ultrasonographic feature in clinical practice in managing Inflammatory Bowel Diseases patients
**2017**	**Poster presentation**	**Bezzio C et al.**	Bowel ultrasound and faecal calprotectin as predictors of response to infliximab in ulcerative colitis
**2023**	**Poster presentation**	**Maeda M et al**	Bowel ultrasound is useful in predicting relapse in patients with Ulcerative Colitis in remission
**2018**	**Not available for download**	**Panes J & Ricart E**	Can we Monitor a Patient with Inflammatory Bowel Disease and Adapt Treatment without Endoscopy?
**2014**	**Foreging languaje**	**M. Prager & C. Büning**	Clinical symptoms, C-reactive protein, calprotectin, MRI or endoscopy? Stragegies for therapy monitoring of IBD
**2013**	**Foreging languaje**	**M. Prager & C. Büning**	Clinical symptoms, C-reactive protein, calprotectin, MRI or endoscopy?: Stragegies for therapy monitoring of IBD
**2021**	**Poster presentation**	**Yogakanthi S et al**	Clinical utility of proactive vedolizumab drug level monitoring: An Australian tertiary hospital experience

**2024**	**Wrong outcome**	**Mohamed EMAA et al**	Combined gray scale ultrasonography and doppler diagnostic tools with strain elastography in assessment of inflammatory bowel disease in pediatrics patients
**2006**	**Wrong outcome**	**Canani RB et al**	Combined use of noninvasive tests is useful in the initial diagnostic approach to a child with suspected inflammatory bowel disease.
**2020**	**Poster presentation**	**Smith R et al**	Comparison of gastrointestinal ultrasound with clinical activity and fecal calprotectin level in the assessment of disease activity in patients with inflammatory bowel disease
**2022**	**Poster presentation**	**Sen G et al**	COMPARISON OF INTESTINAL US WITH CRP, FECAL CALPROTECTIN AND THEIR COMBINATIONS TO PREDICT ENDOSCOPIC REMISSION OF UC
**2022**	**Poster presentation**	**Goodsall TM et al**	Composite assessment using intestinal ultrasound and fecal calprotectin level is accurate in predicting histological activity in ulcerative colitis
**2018**	**Poster presentation**	**Gokli A et al**	Contrast enhanced ultrasound of the bowel in children with suspected inflammatory bowel disease (IBD)
**2015**	**Poster presentation**	**Heerasing N et al**	Correlation of intestinal ultrasound with faecal calprotectin in inflammatory bowel disease
**2021**	**Poster presentation**	**De Voogd F et al**	Decrease In Bowel Wall Thickness At Intestinal Ultrasound Accurately Detects Early Endoscopic Remission And Treatment Response In Ulcerative Colitis Patients On Tofacitinib: A Longitudinal Prospective Cohort Study

**2024**	**Poster presentation**	**Zorzi F et al**	DEFINING<br>DIAGNOSTIC ULTRASONOGRAPHIC PARAMETERS IN ULCERATIVE COLITIS:<br>A LONGITUDINAL PROSPECTIVE MULTICENTER STUDY
**2019**	**Poster presentation**	**Christensen KR et al**	DEVELOPMENT OF AN EVIDENCE-BASED STRATEGY INCORPORATING PATIENT REPORTED OUTCOMES AND PHYSICIANS' PREFERENCES TO MONITOR BIOLOGICAL THERAPIES IN INFLAMMATORY BOWEL DISEASE
**2024**	**Wrong outcome**	**Spyropoulou V et al**	Diagnostic accuracy of multimodal noninvasive follow-up for pediatric ulcerative colitis: A single-center prospective study.
**2024**	**Wrong outcome**	**D'Amico F et al**	Drug Optimization in Patients with Mild-to-Moderate Ulcerative Colitis: A Global Survey.
**2020**	**Poster presentation**	**Smith R et al**	Early assessment with gastrointestinal ultrasound predicts the need for inpatient infliximab therapy in patients hospitalized with a flare of ulcerative colitis
**2022**	**Wrong outcome**	**Sagami S et al**	Early improvement in bowel wall thickness on transperineal ultrasonography predicts treatment success in active ulcerative colitis
**2024**	**Wrong outcome**	**De Voogd F et al [38]**	Early Intestinal Ultrasound Predicts Clinical and EndoscopicTreatment Response and Demonstrates Drug-Specific Kinetics in Moderate-to-Severe Ulcerative Colitis
**2023**	**Wrong outcome**	**Allocca M et al**	Early Intestinal Ultrasound Predicts Long-Term Endoscopic Response to Biologics in Ulcerative Colitis.

**2022**	**Wrong outcome**	**Smith RL et al [28]**	Early sonographic response to a new medical therapy is associated with future treatment response or failure in patients with inflammatory bowel disease.
**2023**	**Wrong outcome**	**Castellano MA et al**	Evaluation of bowel wall flow by color Doppler ultrasound in the assessment of inflammatory bowel disease activity in pediatric patients.
**2024**	**Poster presentation**	**Nagarajan Kvet al**	External validation of Intestinal Ultrasound score: IBUS-SAS with clinical (CDAI), biomarkers and endoscopic scoring system (SES-CD)
**2024**	**Participants are devoid of a diagnosis of UC.**	**Fumery M et al**	Fecal calprotectin, intestinal ultrasound, and their combination for the diagnosis of Crohn's disease
**2011**	**Wrong outcome**	**Aomatsu T et al**	Fecal calprotectin is a useful marker for disease activity in pediatric patients with inflammatory bowel disease.
**2024**	**Wrong outcome**	**Hudson AS et al**	Four intestinal ultrasound scores and bowel wall thickness alone correlated well with pediatric ulcerative colitis disease activity
**2018**	**Wrong outcome**	**Sathananthan D et al**	Gastrointestinal ultrasound in routine inflammatory bowel disease care: Accuracy compared with colonoscopy and fecal calprotectin
**2020**	**Poster presentation**	**Smith R et al**	Gastrointestinal ultrasound performed on admission predicts the need for inpatient infliximab therapy in hospitalised ulcerative colitis patients
**2018**	**Poster presentation**	**Robben S et al**	IBD in children: US or MR-Which one wins?
**2019**	**Participants are devoid of a diagnosis of UC.**	**Bathe AL et al**	Impact of faecal calprotectin measurement on clinical decision-making in patients with Crohn's disease and ulcerative colitis.

**2024**	**Poster presentation**	**Albshesh A et al**	Intestinal ultrasonography accuracy in the evaluation of patients with moderate to severe ulcerative colitis starting infliximab therapy
**2022**	**Poster presentation**	**Couper MR et al**	INTESTINAL ULTRASONOGRAPHY FOR PAEDIATRIC INFLAMMATORY BOWEL DISEASE, DIAGNOSIS, AND MONITORING IN CONJUNCTION WITH SEROLOGICAL TESTS AND FAECAL CALPROTECTIN
**2016**	**Poster presentation**	**Heerasing N et al**	Intestinal ultrasound accurately assesses disease activity in inflammatory bowel disease when compared to faecal calprotectin
**2023**	**Poster presentation**	**Morão B et al**	Intestinal ultrasound as a promising non-invasive monitoring tool in patients with Ulcerative Colitis
**2024**	**Wrong outcome**	**Khan HH et al**	Intestinal ultrasound at diagnosis of pediatric inflammatory bowel disease compared to endoscopy.
**2023**	**Poster presentation**	**Allocca M et al**	Intestinal ultrasound at week 12 predicts long-term endoscopic response to biologics in ulcerative colitis
**2023**	**Poster presentation**	**Saleh A et al**	INTESTINAL ULTRASOUND CAN DETECT ACTIVE INFLAMMATION IN INFLAMMATORY BOWEL DISEASE PATIENTS DESPITE COMBINED CLINICAL AND BIOMARKER REMISSION
**2023**	**Poster presentation**	**Yzet C et al**	Intestinal ultrasound combined to fecal calprotectin is effective to predict endoscopic mucosal healing in ulcerative colitis: A crosssectional study

**2024**	**Poster presentation**	**Bravo A et al**	Intestinal ultrasound correlates with clinical, biochemical, endoscopic and histologic biomarkers in patients with Ulcerative Colitis: a cross-sectional study
**2024**	**Wrong outcome**	**Yzet C et al [37]**	Intestinal Ultrasound, Fecal Calprotectin, and Their Combination to Predict Endoscopic Mucosal Healing in Ulcerative Colitis: A Real-Life Cross-Sectional Study.
**2024**	**Wrong outcome**	**El-Nakeep S et al**	Intestinal ultrasound for follow-up after 24 weeks of biological therapy in inflammatory bowel disease patients: an Egyptian center experience during the COVID-19 pandemic
**2024**	**Poster presentation**	**Maaser C et al**	Intestinal ultrasound for monitoring therapeutic response in patients with ulcerative colitis: results from the TRUST&UC study
**2024**	**Poster presentation**	**Kaniewska M et al**	Intestinal ultrasound for monitoring therapeutic response in patients with ulcerative colitis treated with upadacytynib
**2020**	**Poster presentation**	**De Voogd F et al**	Intestinal ultrasound in ulcerative colitis patients treated with tofacitinib predicts endoscopic outcomes, a longitudinal prospective cohort study
**2020**	**Poster presentation**	**De Voogd F et al**	Intestinal ultrasound throughout pregnancy in inflammatory bowel disease patients, feasibility and reliability of a non-invasive cross-sectional imaging modality
**2024**	**Wrong outcome**	**Pal P et al [30]**	Leveraging existing mid-end ultrasound machine for point-of-care intestinal ultrasound in low-resource settings: Prospective, real-world impact on clinical decision-making

**2024**	**Poster presentation**	**Piazza O Sed N et al**	Maintenance of clinical, biochemical and transmural remission in inflammatory bowel disease patients switching from intravenous to subcutaneous infliximab
**2021**	**Wrong outcome**	**Allocca M et al**	Milan ultrasound criteria are accurate in assessing disease activity in ulcerative colitis: external validation
**2022**	**Poster presentation**	**Allocca M et al**	Milan ultrasound criteria are accurate in assessing endoscopic remission and treatment response in patients with ulcerative colitis.
**2023**	**Wrong outcome**	**Maeda M et al**	Milan Ultrasound Criteria Predict Relapse of Ulcerative Colitis in Remission.
**2024**	**Poster presentation**	**Fremberg Ilvemark J et al**	Monitoring disease activity by intestinal ultrasound predicts biologic treatment persistence in patients with Inflammatory Bowel Disease
**2024**	**Poster presentation**	**Cleveland NK et al**	Monitoring IBD by intestinal ultrasound decreases time to treatment change and time to remission in comparison to conventional management: analysis of patients with IBD on multiple IBD therapies
**2024**	**Poster presentation**	**Krugliak Cleveland N et al**	Monitoring Inflammatory Bowel Disease (IBD) by intestinal ultrasound decreases time to treatment change and time to remission in comparison to conventional management: Analysis of patients with IBD on multiple IBD therapies
**2020**	**Wrong outcome**	**Flanagan E et al**	Monitoring Inflammatory Bowel Disease in Pregnancy Using Gastrointestinal Ultrasonography.

**2019**	**Wrong outcome**	**Maaser C et al [27]**	Monitoring response to anti-tnf therapy in ulcerative colitis patients by gastrointestinal ultrasound: sub-analysis from TRUST&UC
**2021**	**Wrong outcome**	**Cassinotti A et al**	Noninvasive Monitoring After Azathioprine Withdrawal in Patients With Inflammatory Bowel Disease in Deep Remission.
**2019**	**Wrong outcome**	**Cassinotti A et al**	Noninvasive testing in the management of children with suspected inflammatory bowel disease.
**2021**	**Wrong outcome**	**Marin AM et al**	Non-invasive Ultrasonographic Score for Assessment of the Severity of Inflammatory Bowel Disease.
**2023**	**Wrong outcome**	**Hudson AS et al**	Pediatric Patient and Caregiver Satisfaction with the Use of Transabdominal Bowel Ultrasound in the Assessment of Inflammatory Bowel Diseases
**2021**	**Poster presentation**	**Muhammad Nawawi KN et al**	Performance of intestinal ultrasound in comparison with ileocolonoscopy for the disease activity in patients with inflammatory bowel disease
**2023**	**Poster presentation**	**Kumar S et al**	POINT OF CARE INTESTINAL ULTRASOUND IN ADULT PATIENTS WITH INFLAMMATORY BOWEL DISEASE IS ACCURATE AND FEASIBLE
**2021**	**Poster presentation**	**Dolinger M et al**	Point-of-care intestinal ultrasound as a novel, non-invasive biomarker to monitor treatment response to biologic therapy induction in children with inflammatory bowel disease

**2022**	**Wrong outcome**	**Bots S et al**	Point-of-care Intestinal Ultrasound in IBD Patients: Disease Management and Diagnostic Yield in a Real-world Cohort and Proposal of a Point-of-care Algorithm.
**2021**	**Poster presentation**	**Smith R et al**	Predictive value of early gastrointestinal ultrasound after initiation of new therapy for patients with inflammatory bowel disease
**2022**	**Wrong outcome**	**Allocca M et al**	Predictive value of Milan ultrasound criteria in ulcerative colitis: A prospective observational cohort study
**2022**	**Poster presentation**	**Chavannes M**	PROSPECTIVE ASSESSMENT OF BOWEL WALL THICKNESS ON POINT OF CARE INTESTINAL ULTRASOUND IN PEDIATRIC PATIENTS WITH INFLAMMATORY BOWEL DISEASE: A COHORT STUDY.
**2023**	**Wrong outcome**	**Dell'Era A et al**	Relevance of sonographic parameters for inflammatory bowel disease in children.
**2015**	**Poster presentation**	**Dell'Era A et al**	Relevance of ultrasonographic parameters in predicting inflammatory bowel disease in a pediatric population
**2022**	**Poster presentation**	**El-Fekhfakh S et al**	ROLE OF FECAL CALPROTECTIN IN DIAGNOSING INFLAMMATORY BOWEL DISEASE AND IN DETECTING THE DISEASE ACTIVITY
**2022**	**Poster presentation**	**Rueda Sanchez J et al**	Role of gastrointestinal ultrasound in severe ulcerative colitis: A real-life observational study from a tertiary center in Spain

**2024**	**Wrong outcome**	**Otani M et al**	Single measurement of bowel wall thickness using intestinal ultrasonography in children with ulcerative colitis.
**2022**	**Poster presentation**	**Lordache S**	THE ROLE OF INTESTINAL ULTRASOUND IN MONITORING PATIENTS WITH INFLAMMATORY BOWEL DISEASE
**2024**	**Poster presentation**	**Soltan AMK et al**	The Role of Ultrasound in Assessment of Disease Activity in Inflammatory Bowel Disease Patients
**2013**	**Poster presentation**	**Asthana A.K. et al**	The utility of colonic ultrasound in inflammatory bowel disease: A novel australian experience
**2021**	**Wrong outcome**	**Fodor I et al**	The value of abdominal ultrasonography compared to colonoscopy and faecal calprotectin in following up paediatric patients with ulcerative colitis.
**2024**	**Wrong outcome**	**Ollech JE et al [29]**	Tofacitinib is an effective treatment for moderate to severe ulcerative colitis, and intestinal ultrasound can discriminate response from non-response: a pragmatic prospective real-world study
**2023**	**Foreging languaje**	**Vinokurova AV et al**	TRANSABDOMINAL ULTRASOUND COLON DIAGNOSTICS WITH THE PURPOSE OF ASSESSMENT OF THE ENDOSCOPIC ACTIVITY OF ULCERATIVE COLITIS IN CHILDREN
**2019**	**Poster presentation**	**Anda-Eliza L et al**	Ultrasonographic assesment in inflamatory bowel disease patients and faecal calprotectin levels: Emerging tools in monitoring disease

**2019**	**Poster presentation**	**Anda-Eliza L et al**	Ultrasonographic assessment in inflammatory bowel disease patients and fecal calprotectin levels: Emerging tools in monitoring disease activity
**2024**	**Poster presentation**	**Allocca M et al**	ULTRASOUND VERSUS ENDOSCOPY IN ULCERATIVE COLITIS: A HEADTO-HEAD STUDY
**2023**	**Poster presentation**	**Roilidis I et al**	USING BOWEL ULTRASOUND TO ASSESS DISEASE ACTIVITY IN CHILDREN WITH IBD
**2023**	**Wrong outcome**	**Saleh A et al**	Utility of Intestinal Ultrasound in Clinical Decision-Making for Inflammatory Bowel Disease
**2022**	**Poster presentation**	**Huynh Dr H et al**	Validation of UC intestinal ultrasound (UC-ius) index for children with ulcerative colitis
